# Asymmetrical Barkhausen Noise of a Hard Milled Surface

**DOI:** 10.3390/ma14051293

**Published:** 2021-03-08

**Authors:** Mária Čilliková, Anna Mičietová, Robert Čep, Branislav Mičieta, Miroslav Neslušan, Pavel Kejzlar

**Affiliations:** 1 Faculty of Mechanical Engineering, University of Žilina, Univerzitná 1, 01026 Žilina, Slovakia; anna.micietova@fstroj.uniza.sk (A.M.); branislav.micieta@fstroj.uniza.sk (B.M.); miroslav.neslusan@fstroj.uniza.sk (M.N.); 2 Faculty of Mechanical Engineering, VŠB—Technical University of Ostrava, 17. Listopadu 2172/15, 70800 Ostrava, Czech Republic; robert.cep@vsb.cz; 3 Institute for Nanomaterials, Technical University in Liberec, Studentská 1402/2, 46117 Liberec, Czech Republic; pavel.kejzlar@tul.cz

**Keywords:** Barkhausen noise, hard milling, asymmetry

## Abstract

This study is focused on the asymmetrical Barkhausen noise emission of a hard milled surface during cyclic magnetisation. The Barkhausen noise is studied as a function of the magnetising voltage and the hard milled surface is compared with a surface after heat treatment. The asymmetry in the Barkhausen noise emission after hard milling occurs due to the typical “sandwich” structure and the different magnetic hardnesses of the different layers beneath the free surface. Furthermore, this asymmetry is also due to the preferential orientation of the matrix in the direction of the cutting speed and magnetostatic fields, which hinder or favour the premagnetising process.

## 1. Introduction

Hard machining (mainly turning and milling) can be used as a substitute for grinding cycles. The development of machine tools, as well as process technology, has increased the industrial relevance of hard machining [1]. However, the mechanism of chip separation during grinding significantly differs from hard machining. For this reason, the state of the surface produced by these competitive operations is completely different. The main distinctions are as follows [2]: (i) a much longer time period during which higher temperatures penetrate beneath the free surface at grinding (several times greater tool–workpiece contact), (ii) the average stress over the entire contact in grinding is less than in hard milling and (iii) there is deeper penetration of compressive stress in hard milling.

The high heating rates and rapid cooling during hard milling generate the specific state of surface integrity expressed in many terms. Hard milling cycles can suffer from the formation of a white layer (WL) induced at low flank wear *VB* or unexpected catastrophic tool failures. The state of the machined surface after hard milling is mainly a function of the flank wear *VB* and cutting speed. Inserts of high *VB* produce a relatively thick near-surface WL, as well as the corresponding sub-surface heat-affected zone (HAZ) [3]. However, the thickness of the HAZ and WLs after hard milling is about one order of magnitude lower than that induced by grinding. Furthermore, the ratio between the WL and HAZ thickness after hard machining (turning or milling) is much higher compared to grinding [3,4]. Hard turned or milled WLs are denser and more uniform with a severely strained matrix, whereas ground WLs retain their original appearance [2,4,5]. Compared to the bulk, the HAZ produces richer Barkhausen noise emissions (due to tensile stresses, reduced dislocation density and the modification of carbides, i.e., their size, density and morphology), whereas WLs induced by grinding cycles in the near-surface region emit poor Barkhausen noise due to the existence of a higher volume of retained austenite, compressive stresses and very fine grains [2].

Hard milling operations are usually performed at constant cutting conditions. However, the flank wear (associated with mechanical and thermal loads), as well as the corresponding stress and microstructure state, could remarkably vary. A reliable concept for the non-destructive evaluation of surfaces after hard milling cycles is of vital importance, since the surface state expressed in stress or/and microstructure states significantly affects the functionality of the components. The possible concept for non-destructive monitoring of hard milled surfaces based on magnetic techniques (especially Barkhausen noise) has already been discussed [5,6,7].

The magnetic Barkhausen noise (MBN) technique is widely employed for monitoring ground surfaces in real industrial applications. The low MBN values for untouched surfaces contrast against the high MBN emission due to thermal over-tempering during grinding [8,9]. MBN is a product of irreversible discontinuous domain wall motion during cyclic magnetisation. Domain walls interfere with the stress state, as well as microstructural features (such as dislocations, carbides, grain boundaries, non-ferromagnetic particles and so on), which pin domain wall motion [8,9,10]. The high MBN magnitude of over-tempered surfaces after grinding is mainly associated with reduced dislocations and carbide densities (which is thermally initiated by elevated temperatures), which in turn corresponds with the decreased pinning strength of the surface [9].

Very high MBN values and strong magnetic anisotropy can be found after hard milling, despite the low thickness of WLs, as well as the HAZ [5,6,7,11]. Furthermore, cyclic magnetisation initiates a quite specific (asymmetrical) character of MBN bursts, especially at lower magnetising voltages. This phenomenon has not been explained yet. Thus, this study deals with this aspect and compares surfaces after heat treatment and consecutive hard milling.

## 2. Materials and Methods

The experimental study was carried out on bearing steel 100Cr6 heat treated at a hardness of 61 ± 1 HRC. Heat treatment of the samples was carried out in industrial conditions. Samples with the dimensions of 70 mm × 30 mm × 25 mm were quenched from an austenitising temperature of 840 °C in an oil of temperature 60 °C and tempered afterwards at a temperature of 140 °C for 2 h. Hard milling was carried out by the use of a 050Q22-12M 262489 milling cutter of diameter Ø 50 mm with two inserts of flank wear *VB* = 0.05 mm. A cutting depth of *a_p_* = 0.25 mm, a feed speed of *v_f_* = 112 mm·min^−1^ (the feed direction corresponds to the axial direction on the milled surface) and a cutting speed of *v_c_* = 78.5 m·min^−1^ (the cutting speed direction corresponds to the tangential direction on the milled surface) were used. More details regarding the process kinematics and MBN measurements can be found in previous publications [4,5,6]. MBN was measured by the use of RollScan 300 (Stresstech, Jyväskylä, Finland) and software µScan (version 5.4.1) (magnetising voltage in the range of 4 to 16 V, a magnetising frequency of 125 Hz, ten bursts and a frequency range of MBN signal from 10 to 1000 kHz). MBN refers to the rms (effective) value of the signal. To reveal the microstructural transformations induced by hard milling, 10-mm-long pieces were prepared for SEM (UHR Carl Zeiss Ultra Plus, Jena, Germany) and metallographic observations (etched by 5% Nital for 8 s). The microstructure was observed in the direction of the cutting speed. The samples were demagnetised before the measurements.

## 3. Results of Experiments

**Figure 1:**
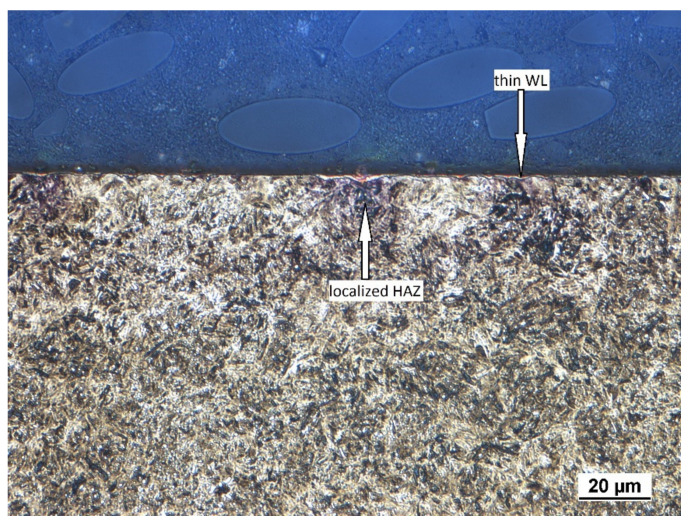
Thin and discontinuous white layer (WL) after hard milling with localised spots of thermally softened dark spots beneath the WL.

As mentioned above, the hard milling cycle produces a WL in the near-surface region, followed by the HAZ in the deeper sub-surface layers. Figure 1 illustrates that the WL produced by the insertion of low *VB* (0.05 mm) is very thin and discontinuous. This layer appears white under the metallographic observations due to its high resistance against etching. Moreover, the HAZ (which appears dark) is also localised and its remarkable dark spots can be found just below the WL position, in which the WL thickness randomly increases in thickness.

The presence of a WL in such a surface indicates that the WL is a product of the temperature cycle when the hard milled surface is heated over the austenitising temperature, followed by rapid self-cooling, which in turn results in re-hardening of the near-surface layer. The previous study of Guo [2] indicated that the hardness of the re-hardened WL layer after is higher than the bulk. Alternatively, the deeper HAZ represents a softened region [2]. The HAZ, as the sub-surface layer, is the region in which the elevated temperature does not exceed the austenitising temperature and the hard milling cycle initiates structure over-tempering only. The HAZ is therefore a tempered zone of reduced resistance against etching.

**Figure 2:**
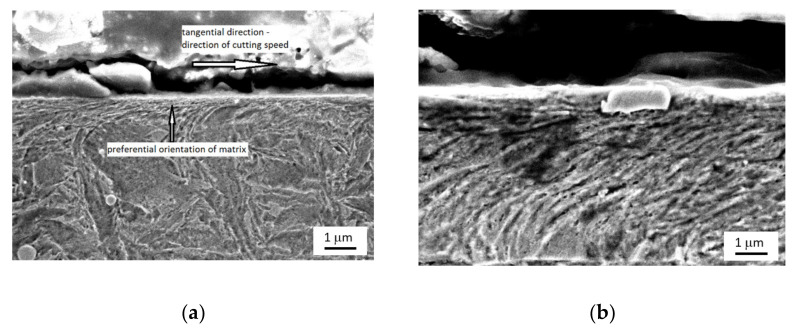
SEM pictures of preferential orientation of martensite matrix after hard milling. (**a**) Macro view, (**b**) detail.

The SEM images of the surface depicted in Figure 2 show that the martensite matrix in the near-surface layer is preferentially oriented in the direction of the cutting speed and becomes tilted at a certain angle against the free surface with increasing depth. More remarkable visualisation of *VB*, the HAZ and the preferential orientation of the martensite matrix can be found for surfaces produced by inserts of higher *VB*. Such figures can be found in previous reports [4,5,7].

**Figure 3:**
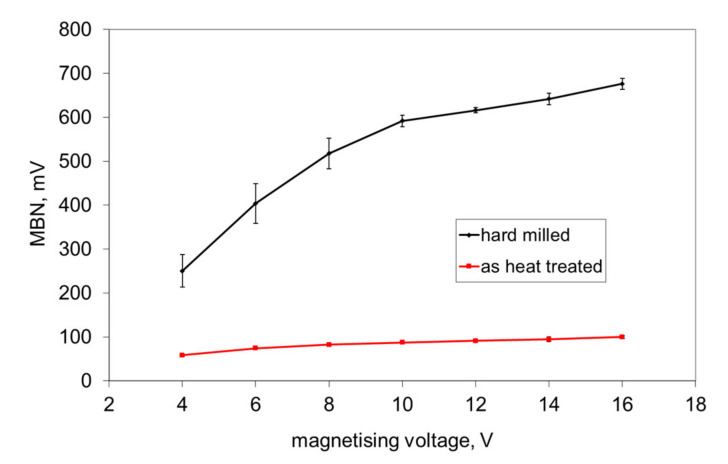
Magnetic Barkhausen noise (MBN) as a function of the magnetising voltage.

The preferential orientation of the martensite matrix after hard milling is very important and explains the high MBN emission in the tangential direction (which is usually not expected for bearing steel of hardness 62 HRC and the corresponding high magnetic hardness), see Figure 3. As reported [5,6], the main reason can be viewed as the cutting temperature exceeding the Curie temperature needed to disturb the domain configuration of the ferromagnetic steel. The domain configuration of the near surface during heating is disturbed and the new domain alignment is configured during rapid cooling. Domains are not random, but preferentially oriented in the direction of the cutting speed (tangential direction). It has already been reported that the domain alignment follows the preferential matrix orientation in hard machining cycles [12].

**Figure 4:**
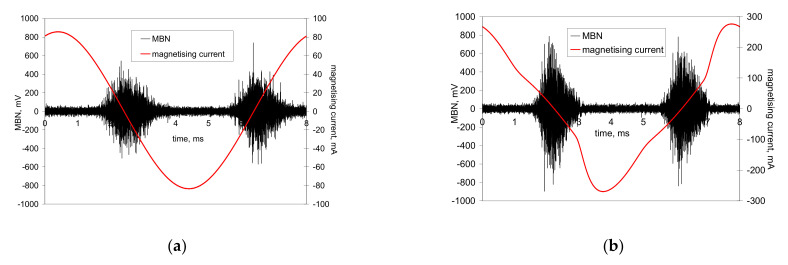
MBN signals after heat treatment. (**a**) Magnetising voltage of 6 V; (**b**) magnetising voltage of 14 V.

The preferential orientation of domain walls in the direction of the cutting speed at the expense of the axial direction results in remarkable magnetic anisotropy after hard milling [4,5,6,7]. Alternatively, it remarkably affects the appearance of MBN bursts due to the specific character of the domain wall motion during cyclic magnetisation. Conventional MBN bursts emitted by the isotropic surface, such as that after heat treatment, are illustrated in Figure 4. As soon as the first weak MBN pulse in the time scale is detected, the amplitude of MBN pulses gradually increases with time and the corresponding strength of the magnetic field. This amplitude attains a maximum and decreases afterward. An explanation of such behaviour is connected to domain walls’ irreversible and discontinuous motion and the corresponding electromagnetic pulses.

Matrix precipitates, grain boundaries and free surfaces usually produce magnetic dipoles. Then, the secondary domain structure in the form of closure (reversed) domains can be found at the boundary of precipitates (or other non-ferromagnetic particles) and the matrix, on the free surface and near grain boundaries (to reduce or diminish the magnetostatic energy). Such domains confirm the observations of Batista [13]. Closure domains decrease in size with an increasing magnetic field; however, some of them could retain the domain structure despite very high magnetic fields being employed. As soon as the magnetic field is reversed, the domain subsequent growth occurs in the opposite direction and domain wall motion is preferentially initiated on the already presented closure domains. It is worth mentioning that the low amplitude of MBN pulses in this stage of the magnetisation process is mainly associated with the motion of 90° domain walls. The lower MBN amplitude produced by 90° domain walls is driven by the low misorientation of 90° domain walls and their reduced length.

**Figure 5:**
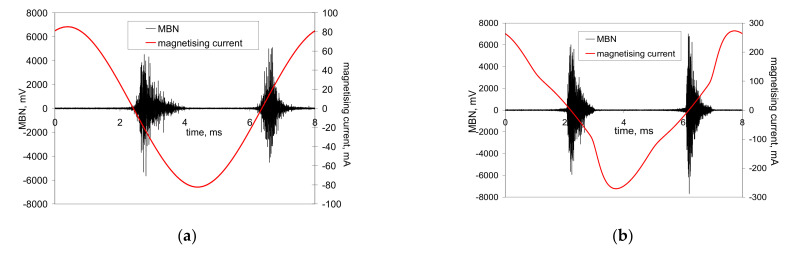
MBN signals after hard milling. (**a**) Magnetising voltage of 6 V; (**b**) magnetising voltage of 14 V.

Conventional MBN bursts, such as those illustrated in Figure 4, occur in all cases when the magnetic field for domain nucleation *H*_n_ is less than the magnetic field needed for their growth *H*_g_. Alternatively, the very high MBN emission for the hard milled surface is due to *H*_n_ > *H*_g_. The high *H*_n_ is due to the strong surface texture after hard milling (the small misorientation of the neighbouring grains decreases the density of magnetic dipoles). The domains in the near-surface region stay aligned parallel with the machined surface. Cyclic magnetisation only changes their alignment to the opposite direction. MBN in this layer occurs in the form of a single massive MBN event (or a few MBN jumps). The specific mechanism of the domain wall motion is exhibited in Figure 5b, in which an abrupt and immediate (no gradual) increase in MBN magnitude (very short rise time) can be found during magnetisation.

Figure 4 and Figure 5 show two consecutive MBN bursts (one magnetising cycle). The first half represents the MBN burst during the descending magnetising current (negative burst), whereas the second one, referred to as positive, is the MBN burst during the ascending magnetising current. Figure 4 and Figure 5b show that both bursts are more or less similar. Conversely, the consecutive MBN bursts in Figure 5a are remarkably different. In other words, the premagnetisation process (realignment of domain walls) is different in the direction of the cutting speed, compared to the opposite direction. However, such behaviour can be found especially at lower magnetising voltages. As soon as the magnetising voltage increases, the differences between the consecutive bursts disappear.

**Figure 6:**
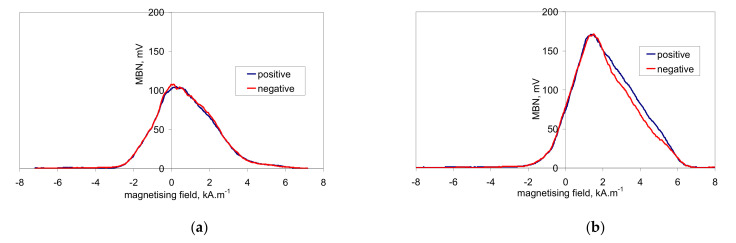
MBN envelopes after heat treatment. (**a**) Magnetising voltage of 6 V; (**b**) magnetising voltage of 14 V.

**Figure 7:**
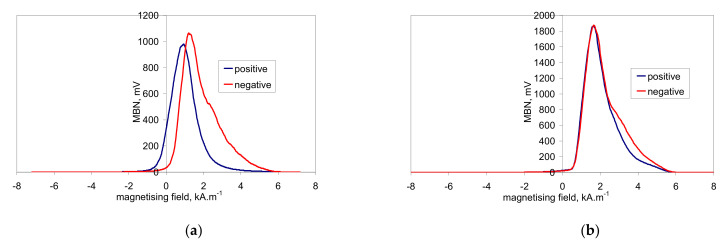
MBN envelopes after hard milling. (**a**) Magnetising voltage of 6 V; (**b**) magnetising voltage of 14 V.

**Figure 8:**
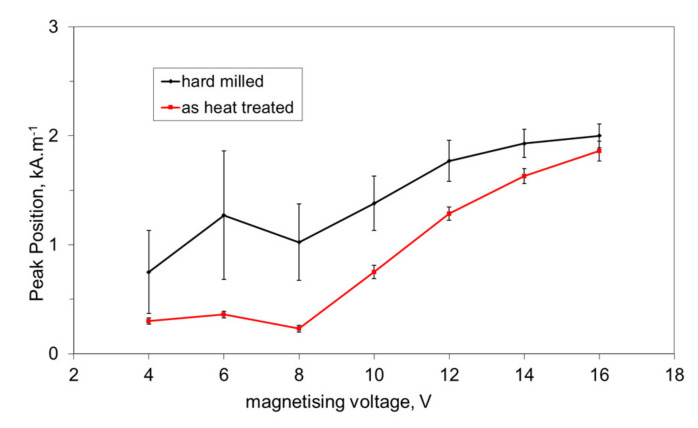
Peak position of MBN envelopes as a function of the magnetising voltage.

This asymmetry during cyclic magnetisation can also be found in MBN envelopes, as illustrated in Figure 6 and Figure 7 (MBN produced by the sensor coil was subtracted from the MBN envelopes). The differences in two consecutive MBN envelopes more or less correspond to the differences in MBN shape and position (expressed in terms of magnetising field *H*). Both MBN envelopes coincide for the surfaces after heat treatment, as well as after hard milling at a magnetising voltage of 14 V (some differences can be found in the descending part of the MBN envelopes). As soon as the magnetising voltage is decreased below 10 V, remarkable differences in the MBN envelopes can be seen (expressed in terms of MBN peak height and position). As can be seen in Figure 7a, the MBN burst during the descending magnetising current is shifted to the higher magnetic field, compared to the MBN burst during the ascending magnetising current. Furthermore, this figure also demonstrates that the effective (rms) value of MBN differs within the one magnetising cycle (corresponding to the different peak heights of the MBN envelopes). This aspect confirms Figure 3, in which the maximum and minimum MBNs within one magnetising cycle are indicated (instead of error bars usually referring to the standard deviation of repetitive measurements). This figure shows that differences between the MBN values are much higher at lower magnetising voltages than for higher ones. Such behaviour can also be found when considering the peak position of the MBN envelope, as illustrated in Figure 8. This figure also shows that the magnetic strength of the WL is more than the untouched surface (after heat treatment).

The exact evaluation of MBN asymmetry during cyclic magnetisation is based on Equation (1) as follows:

(1) $$diff=\;\left|\frac{\sum_{events}\int mV^{2}dH_{pos}\;-\;\sum_{events}\int mV^{2}dH_{neg}\;}{\sum_{events}\int mV^{2}dH_{pos}\;+\;\sum_{events}\int mV^{2}dH_{neg}\;}\right|$$

where *mV* is the induced voltage in the pick-up coil originating from MBN jumps and *diff* refers to the ratio between the sum of areas in which both MBN envelopes do not coincide with the area of both MBN envelopes (separately).

**Figure 9:**
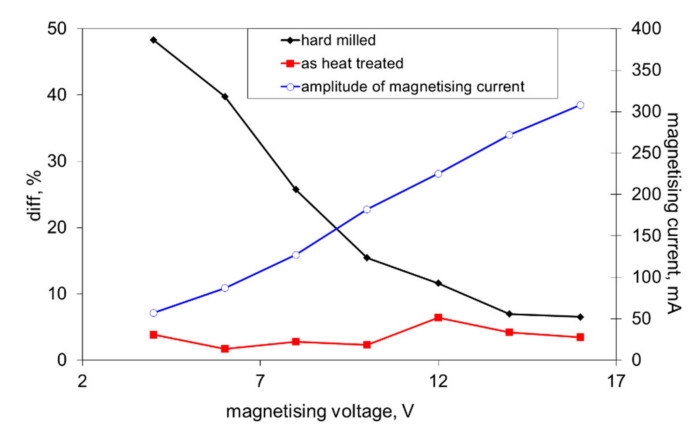
Evolution of the magnetising current and *diff* versus the magnetising voltage.

Figure 9 shows that this difference is kept nearly constant for the surface after heat treatment, whereas *diff* steeply increases along with the decreasing magnetising voltage for the surface after hard milling.

## 4. Discussion of Results

One might argue that MBN bursts would occur in the form of a rectangular shape in the time scale (or the scale of the magnetic field). It should be noticed that the typical preferential orientation of the matrix after hard milling can be found only in the limited thickness of the near-surface region (a few µm), whereas the estimated MBN sensing depth in this case is about 40 µm. For this reason, the MBN burst, as illustrated in Figure 5b, contains MBN pulses from the preferentially oriented matrix, from the deeper HAZ and the untouched bulk.

**Figure 10:**
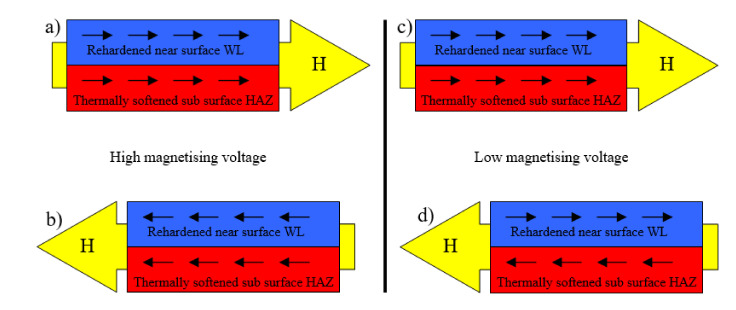
Schematic drawings illustrating the pseudo-saturation states after hard milling at different magnetising voltages. (**a**) High voltage and ascending field, (**b**) high voltage and descending field, (**c**) low voltage and ascending field, (**d**) low voltage and descending field.

The hard milling cycle produces the typical “sandwich” structure. The re-hardened near-surface WL is followed over tempered or untouched deeper layers. Hard milling should be considered as a steep gradient structure in which stress and structure dramatically alter along with increasing depth beneath the free surface due to particularly rapid self-cooling rates. It is also worth mentioning that mechanical hardness usually corresponds to the magnetic hardness of a body. For this reason, higher magnetic fields are needed to unpin the harder near-surface WL, as opposed to the deeper softer and/or untouched layer—see also Figure 8 (it is estimated that the magnetic hardness of the HAZ expressed in terms of peak position is less than the bulk, with the bulk corresponding to the structure with heat treatment). The consequence of such a statement results in different processes of premagnetisation under different magnetising voltages, as Figure 10 illustrates. The magnetising voltage directly affects the amplitude of the magnetising current (see Figure 9) and the corresponding rate of d*H*/d*t*. Furthermore, it should be noticed that the magnetising voltage also affects the distribution of the magnetic field and its strength in the different layers beneath the free surface. Therefore, the high magnetising voltage unpins and switches the magnetisation of the near-surface WL, as well as the deeper thermally softened HAZ (or untouched bulk)—see Figure 10a,c. As soon as the magnetising voltage is turned down, the magnetic field becomes weaker and switches the magnetisation on the softened HAZ (or untouched bulk), whereas the domain alignment and the corresponding domain wall alignment of the near-surface re-hardened WL remain untouched. Such behaviour corresponds to the observation of Rivas [14]. Rivas et al. investigated monolithic amorphous bilayer ribbons of Fe_73.5_Nb_3_Si_13.5_B_9_Cu_1_/Fe_74.5_Nb_3_Si_13.5_B_9_. The authors separated the individual contributions of each layer into the global magnetic behaviour and analysed the magneto-coupling between them. The findings of this study match well the magnetic behaviour of the hard milled surface.

Figure 10b,d show that the MBN emission at lower magnetising voltages and the corresponding weaker magnetic fields should be attributed mostly to the HAZ (and bulk) and strongly depends on the magnetic history of the re-hardened WL. When the WL has been previously oriented by a sufficiently strong premagnetising field, the weaker magnetising field initiates only premagnetisation of the deeper HAZ (and bulk). Asymmetry in MBN under the weaker magnetising fields is due to magneto-coupling between the re-hardened WL (which stays unpinned) and softer HAZ (and bulk). As reported by Rivas, the magnetostatic field that the WL produces in HAZ is, for instance, negative (no matter whether the applied field is positive or negative). This hinders the positive saturation and favours the negative one.

To gain deeper insight into the magnetisation process, especially the nucleation process associated with 90° domain walls and the massive MBN pulses originating from 180° domain walls in the preferentially oriented near-surface WL, further analyses were carried out. Martines-Ortiz et al. [14] reported that MBN events nearby the main peak in the MBN envelope are associated with the pure 180° domain walls’ motion. The authors showed that the width of this region (*dH_180_*) is 25% of all the magnetic fields (*dH_e_*), in which any MBN events occur, and named this region *R^180^* [15]. The width (and position) of this region is defined as the MBN envelope maximum ±12.5% of *dH_e_*. The region (*dH_90_*) between the first point in which the MBN envelope grows above the background noise and the *R^180^* region is attributed to the nucleation (and the motion), mainly reversed (90° BWs), domains. This region can be named *R^90^* hereafter. The MBN energy in a certain region (defined by the width *dH*) can be calculated using Equation (2).

(2) $$E\;=\;\underset{events}{\sum }\int mV^{2}dH$$

It should be noted that the exact evaluation of the different regions could be debatable. In particular, the boundary between *dH_90_/dH_180_* should be considered as a region in which mixed MBN motion could be expected (motion of 180° and 90° domain walls), as reported in a previous study [16]. With a simplified model of *dH_90_* and *dH_180_*, the distribution on the MBN envelope, such as that reported in [15], provides deeper insight into MBN.

**Figure 11:**
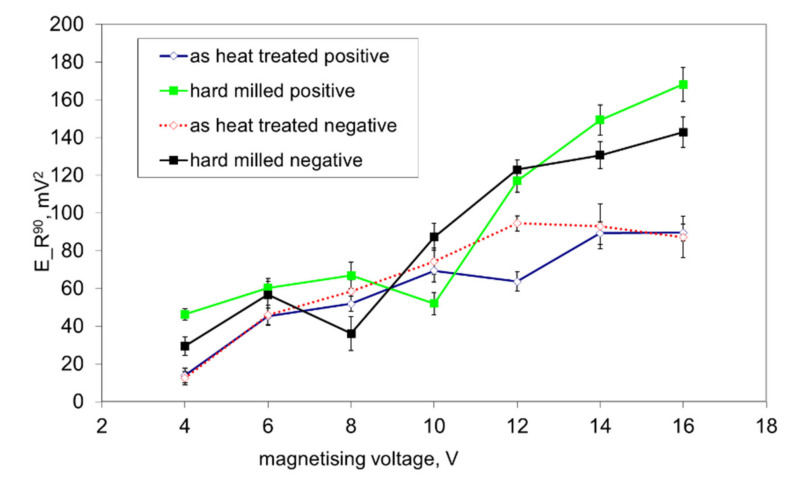
*E_R^90^* as a function of the magnetising voltage.

**Figure 12:**
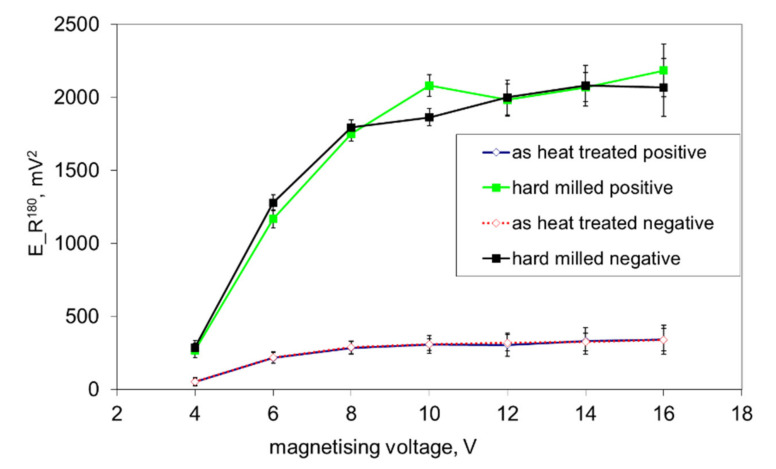
*E_R^180^* as a function of the magnetising voltage.

Figure 11 shows that both surfaces exhibit nearly the same *E_R^90^* at lower magnetising voltages (mainly originating from the HAZ and bulk in the case of the hard milled surface), whereas a higher *dH_90_/dH_180_* can be found for magnetising voltages in the range of 12 to 16 V (as a contribution to the nucleation processes in the WL). This figure shows that the nucleation processes in the WL at higher magnetising voltages still can be detected despite the preferential orientation of the WL. Manh [17] reported that the number and the size of MBN events in this magnetisation stage are associated mainly with the presence of magnetic free poles related to the misorientation between the grains and therefore to the misorientation angle distribution of the surface. However, due to the preferential orientation of the matrix in the near-surface region, the main source of closure domains (in this particular case) and the corresponding 90° domain walls’ motion can be viewed mainly in the presence of carbides in this very thin WL, as Figure 2b depicts. Figure 12 shows that the preferential orientation of the WL, the associated preferential orientation of 180° domain walls into the direction of magnetisation and the corresponding very strong MBN pulses result in very high *E_R^180^*, as opposed to the surface after heat treatment. This figure also shows that *E_R^180^* remarkably decreases with decreasing magnetising voltage, since the contribution of the near-surface re-hardened WL becomes weaker.

The decrease in *E_R^180^* in Figure 12 (along with the decreasing magnetising voltage) is gradual and there is a no clear boundary which indicates that the WL is not unpinned and cannot be detected (see also Figure 3, Figure 8 and Figure 9). The important difference between the bilayer ribbon (reported in [14]) and the hard milled surface is in the clear boundary between the softer and harder layers. The bilayer ribbon exhibits a clear boundary between two different layers, whereas the hard milled surface should be considered as a gradient structure, as mentioned above. On this basis, the magnetic hardness and/or preferential orientation correspond to the microstructure and stress gradient. As Figure 2b illustrates, the near-surface region is nearly parallel with the cutting direction. However, the matrix becomes tilted with the increasing angle against the free surface with increasing depth beneath the free surface. This means that the contribution of the different layers beneath the surface along with increasing (or decreasing) magnetising voltage would alter. Finally, it can be noticed that the remarkable asymmetry in the MBN envelopes occurs during the uniaxial tensile test at certain plastic strains, as it was reported in [18,19]. However, the non-homogeneity in the grains’ plastic straining takes a major role in this particular case. Furthermore, the contribution of stresses should be considered as well [20,21].

## 5. Conclusions

The main findings of this study can be summarised as follows:

- The preferential orientation of the near-surface matrix into the cutting speed direction takes a major role in the high MBN emission after hard milling;
- The remarkable asymmetry in the consecutive MBN bursts and the corresponding MBN envelopes is due to magnetic coupling between the hard near-surface WL and underlying softer HAZ;
- The asymmetry in MBN bursts is a function of the magnetising field and tends to disappear at higher d*H*/d*t*;
- The asymmetry of the consecutive MBN bursts mainly affects the positions of MBN envelopes and the corresponding peak positions, whereas the effective values of the raw MBN signals remain nearly unchanged.

Hard milled components are subjected to the non-destructive monitoring of their surfaces via the MBN technique. The possible concepts have already been discussed. Implementation of the MBN technique in real industrial applications usually represents the MBN calibration when MBN values refer to the certain microstructural alterations. The second task is associated with the evaluation of the MBN threshold as a critical value for the approved or unacceptable surface state. The hard milling process represents a variety of component hardnesses, associated sensors, MBN techniques and magnetising conditions. It should be kept in mind that MBN values could remarkably vary (at certain magnetising conditions) due to asymmetry in the premagnetising process such as those reported in this study. As a consequence, these MBN values can attain or exceed an MBN threshold despite an acceptable state of surface integrity after hard milling.

## Data Availability

The raw data required to reproduce these findings cannot be shared easily due to technical limitations (especially MBN raw signals are too large due to very high sampling frequency). However, authors can share the data on any individual request (please contact the corresponding author by the use of its mailing address).
